# Temporal and anatomic determinants of central-line–associated bloodstream infection risk in a tertiary PICU: a 3-year time-to-event and competing-risk analysis

**DOI:** 10.3389/fped.2026.1763433

**Published:** 2026-06-12

**Authors:** Khouloud A. Alsofyani, Ibrahim H. Muzaffar, Abeer A. Alnajjar, Mohammed Shahab Uddin

**Affiliations:** 1 Department of Pediatrics, Faculty of Medicine, King Abdulaziz University, Jeddah, Saudi Arabia; 2 Pediatric Intensive Care Unit, King Abdulaziz University Hospital, Jeddah, Saudi Arabia; 3 Clinical Skills and Simulation Center, Faculty of Medicine, King Abdulaziz University, Jeddah, Saudi Arabia; 4 Infectious diseases unit, Department of Pediatrics, Faculty of Medicine, King Abdulaziz university, Jeddah, Saudi Arabia; 5 Department of Pediatrics, Ministry of National Guard Health Affairs, Dammam, Saudi Arabia; 6 King Abdullah International Medical Research Center, Riyadh, Saudi Arabia; 7 King Saud bin Abdulaziz University for Health Sciences, Riyadh, Saudi Arabia

**Keywords:** central line-associated bloodstream infection, central venous catheter, pediatric intensive care, catheter dwell time, competing-risk analysis, survival analysis, catheter insertion site, parenteral nutrition

## Abstract

**Background:**

Central line–associated bloodstream infections remain a major cause of preventable morbidity in pediatric intensive care units, yet most prevention strategies treat infection risk as static over catheter dwell time.

**Objective:**

We aimed to characterize the temporal and anatomic determinants of central line–associated bloodstream infection in a tertiary pediatric intensive care unit using patient–day survival data.

**Methods:**

In this retrospective cohort study, 300 central venous catheter insertions in children admitted to a tertiary pediatric intensive care unit between January 2020 and December 2022 were screened for eligibility. The daily risk of first central line–associated bloodstream infection was modelled using discrete-time pooled-logistic regression with ridge regularization, incorporating baseline covariates, catheter dwell time (0–14, 15–22, and ≥23 days), time-varying clinical markers lagged by 24 hours, and catheter insertion site (femoral, internal jugular, subclavian). Fine–Gray subdistribution hazard models accounted for competing events (catheter removal, discharge, or death). Model performance was evaluated using bootstrap-corrected Harrell's C-index and calibration plots.

**Results:**

After exclusions, 270 non-tunneled central venous catheters contributed 4,304 catheter-days. Over 4,304 catheter-days, 13 central line–associated bloodstream infections occurred (incidence 3.0 per 1,000 catheter-days). Compared with dwell time less than 14 days, adjusted odds of infection were 4.33 (95% CI 1.89–9.92) for days 15–22 and 11.71 (95% CI 3.22–42.62) for dwell time of 23 days or more. Right internal jugular insertion was associated with lower adjusted odds relative to femoral access (adjusted odds ratio 0.34; 95% CI 0.14–0.81). Fever in the preceding 24 hours was associated with a threefold higher odds of infection (adjusted odds ratio 3.00; 95% CI 0.94–9.52). The model demonstrated good discrimination (bias-corrected C-index 0.78; 95% CI 0.70–0.86) and acceptable calibration.

**Conclusion:**

In this tertiary pediatric intensive care unit, central line–associated bloodstream infection risk was strongly phase-dependent, rising sharply after the second week of catheter dwell and particularly beyond 23 days, and was lower with right internal jugular than femoral insertion. These temporal and anatomic findings support time-triggered line-necessity reviews and careful consideration of insertion site within the broader clinical context when seeking to reduce infection risk in critically ill children.

## Background

Central line–associated bloodstream infections (CLABSI) represent a significant and enduring threat to patient safety in the pediatric intensive care unit (PICU). Despite concerted international efforts to reduce their incidence through standardized prevention bundles, these infections remain a leading cause of preventable harm in critically ill children (1, 2). The clinical and economic consequences are substantial, contributing to prolonged lengths of ICU and hospital stay, increased healthcare expenditures, and a considerable burden of attributable morbidity and mortality (3–5).For this vulnerable population, a CLABSI is not merely a complication but a pivotal event that can markedly alter the clinical trajectory (5, 6).

A substantial body of evidence has identified several key modifiable risk factors that predispose critically ill children to CLABSI (7–11). The administration of total parenteral nutrition (TPN) is a consistently cited risk, thought to increase infection rates both by providing a nutrient-rich medium for microbial growth and by necessitating frequent catheter access (12–17). Patient-related factors, particularly underlying acuity and systemic inflammation, also play a crucial role. In febrile children, higher presenting temperature and abnormal leukocyte counts have been associated with increased odds of CLABSI, and in PICU populations new-onset fever is frequently due to device-related infections, including CLABSI (18, 19). Furthermore, catheter-specific characteristics are critically important. The anatomic insertion site remains a point of emphasis in prevention guidelines, with data suggesting that femoral catheterization may carry a higher risk compared with subclavian or internal jugular sites in certain pediatric populations (20–22). Understanding how these established risk factors interact over time is fundamental to refining and targeting prevention strategies (10, 11).

Among the various modifiable risks, the duration of catheterization, or dwell time, is a universally recognized driver of CLABSI, with each additional day demonstrably increasing the cumulative risk of infection (9, 22–25). As a central line remains *in situ*, the risk of intraluminal colonization and subsequent biofilm formation escalates, creating a nidus for infection that becomes increasingly difficult to eradicate (26). This direct, time-dependent relationship between longer dwell times and higher infection rates has been well documented across pediatric populations, including in the PICU (9, 23–25). Consequently, a cornerstone of major prevention guidelines is the daily assessment of catheter necessity and the prompt removal of any line that is no longer essential, a practice aimed at minimizing exposure time (24, 27, 28).

However, the current understanding of how dwell time translates into infection risk remains surprisingly simplistic. Most existing studies have treated catheter duration as a static or linear covariate in conventional regression models, often relying on arbitrary cutoffs that fail to capture the true, dynamic nature of daily infection hazard (29, 30). This approach provides limited clinical guidance beyond the general advice to remove catheters early. Consequently, precise, data-driven delineation of a potential relative “safe window”—and the specific point at which CLABSI risk begins to accelerate—remains a critical knowledge gap for bedside clinicians. By employing more sophisticated time-to-event methodologies, such as competing-risk analysis and the estimation of daily cause-specific hazards, it becomes possible to model this temporal relationship with greater accuracy (25, 31, 32). To date, very few pediatric studies have explicitly examined CLABSI risk at the level of patient–days while simultaneously treating catheter removal, PICU discharge, and death as competing events, leaving clinicians with limited evidence to guideline-stewardship decisions beyond broad dwell-time thresholds (9–11, 25).

Therefore, this study sought to quantify the temporal dynamics of CLABSI risk in a tertiary PICU over three years using patient–day survival data. We applied discrete-time ridge regression and Fine–Gray competing-risk models to delineate time-specific infection phases, to assess the independent effects of anatomical site and modifiable covariates (fever, TPN, leukocytosis), and to derive internally validated absolute risk estimates that can directly inform clinical decision-making.

## Materials and methods

### Study design and setting

We conducted a retrospective observational cohort study in the multidisciplinary pediatric intensive care unit (PICU) of a tertiary referral academic hospital. The PICU admits medically and surgically ill children from infancy through adolescence and provides advanced organ support, including invasive mechanical ventilation, vasoactive infusions, renal replacement therapy, and continuous monitoring. All central venous catheter (CVC) insertions and PICU admissions between 1 January 2020 and 31 December 2022 were screened for eligibility. The unit follows standardized central-line insertion and maintenance bundles, with CLABSI surveillance performed according to institutional infection-prevention protocols and CDC/NHSN definitions. The PICU followed a standardized central-line insertion and maintenance bundle throughout the study period. Core insertion practices included hand hygiene, maximal sterile barrier precautions, and appropriate skin antisepsis according to pediatric institutional policy. Core maintenance practices included sterile occlusive dressing application with routine assessment and scheduled dressing change or earlier replacement if the dressing became loose, wet, or visibly soiled, disinfection of catheter hubs/connectors before each access, minimization of unnecessary line manipulation, and daily review of line necessity with prompt removal when the catheter was no longer clinically indicated. In our PICU, line necessity was also reviewed during daily goal-directed rounds, during which the care team explicitly addressed whether the central line remained necessary.

Compliance with the central-line bundle was monitored through routine electronic documentation review as part of institutional infection-prevention surveillance. During the study period, documented overall bundle compliance remained consistently high, ranging from 98% to 99%. All non-tunneled central venous catheters, including femoral, jugular, and subclavian lines, were managed under the same standardized institutional bundle framework. However, the retrospective analytic dataset did not contain sufficiently granular catheter-level or site-specific compliance data to permit direct comparison of adherence between femoral and non-femoral lines or separate modelling of insertion-bundle and maintenance-bundle compliance. This study involved human participants and was conducted as a retrospective review of pediatric medical records. The protocol conformed to the ethical guidelines of the 1975 Declaration of Helsinki and was approved by the Unit of Biomedical Ethics Research Ethics Committee, Faculty of Medicine, King Abdulaziz University, Jeddah, Saudi Arabia [NCBE registration No. HA-02-J-008; Reference No. 267-24, Non-Intervention (Retrospective Record Review)]. Owing to the retrospective, non-interventional nature of the study and the use of de-identified data, the requirement for informed consent from individual patients was waived by the ethics committee. Patient confidentiality was strictly maintained, and no directly identifiable patient information is presented in this manuscript.

### Participants and central line characteristics

**Figure 1:**
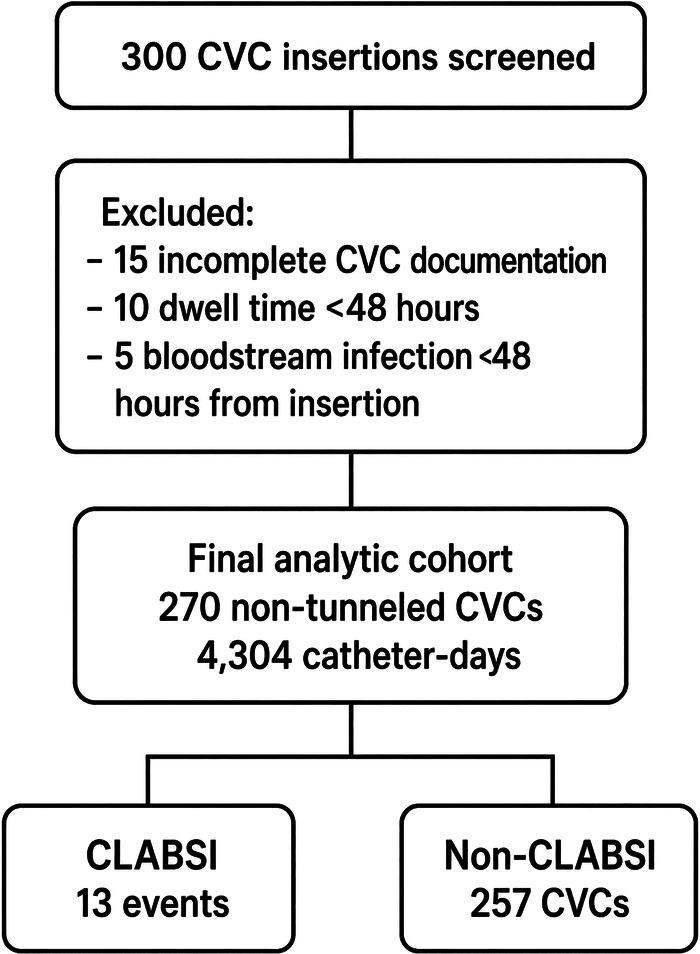
Flow diagram of cohort selection. Among 300 pediatric intensive care unit (PICU) central venous catheter (CVC) insertions screened (January 2020–December 2022), 30 were excluded (15 for incomplete documentation, 10 for dwell time <48 h, and 5 for early bloodstream infection). The final analytic cohort comprised 270 non-tunneled CVCs contributing 4 304 catheter-days, with 13 confirmed central line–associated bloodstream infections (CLABSIs), yielding a crude rate of 3.0 per 1,000 line-days. The diagram summarizes screening, exclusions, and classification into CLABSI and non-CLABSI groups.

All children aged 1 month to 14 years admitted to the PICU who had a central venous catheter (CVC) inserted between 1 January 2020 and 31 December 2022 were eligible for screening. We identified 300 CVC insertions during the study period. Catheters were included if they were non-tunneled, inserted in the PICU or operating room, and remained *in situ* for at least 48 h. We excluded catheters if there was evidence of bloodstream infection at or within 48 h of insertion, if the infection episode was judged to be secondary to a non–catheter source (including mucosal barrier injury), or if key line-related or outcome data were incomplete. After applying the eligibility criteria described above, the final analytic cohort was assembled and each catheter episode was followed from insertion until the earliest of central-line–associated bloodstream infection, catheter removal, discharge, death, or 28 days of dwell. The selection process and cohort derivation are described in the Results section (Figure 1). Each CVC episode was analyzed at the line level. The analytic unit was the catheter episode rather than the individual child. When the same child was readmitted on a separate occasion and required a new central venous catheter, that later catheterization was counted as a separate observation because it represented a distinct admission and clinical episode. Accordingly, the final analytic cohort comprised 270 CVC episodes, and the number of unique children may have been lower than the number of catheter episodes. For each catheter, we recorded patient demographics and comorbidities, primary admission diagnosis, indication for central access, and organ support at insertion (mechanical ventilation, vasoactive infusions, renal replacement therapy). Catheter-specific variables included anatomic insertion site (femoral, right internal jugular, left internal jugular, subclavian), side (right/left), lumen number, and whether the line was placed under ultrasound guidance. Although insertion site and catheter characteristics were recorded, the urgency of catheter placement (emergent vs. elective) was not uniformly documented in the retrospective source records in a manner suitable for reliable analysis. This limitation applied to both femoral and non-femoral catheters and therefore the urgency of insertion could not be incorporated as a covariate in the present study. Dwell time was calculated in whole days from the date and time of insertion to the date and time of removal or first CLABSI event.

### Outcome definition

The primary outcome was the occurrence of a first central line–associated bloodstream infection (CLABSI) for each catheter episode during the PICU stay. Potential CLABSI events were adjudicated by the institutional infection prevention team according to CDC/NHSN surveillance definitions specified in the Patient Safety Component Manual, Chapter 4: Bloodstream Infection Event, using the annual manual version in effect for the relevant event year (2020, 2021, or 2022), requiring a laboratory-confirmed bloodstream infection not attributed to an alternative primary source, with the central venous catheter in place for more than 2 calendar days on the date of event and present on the date of event or the day before. Episodes classified as mucosal barrier injury–related bloodstream infections or secondary bloodstream infections from a non–catheter source were not considered CLABSIs and were excluded.

Potential CLABSI events were identified through routine infection-prevention surveillance and review of positive blood cultures, with final classification made by the hospital infection control team based on standardized CDC/NHSN definitions. For time-to-event analyses, follow-up for each catheter began at insertion and continued until the earliest of first CLABSI, catheter removal, PICU discharge, death, or 28 days of dwell, which was the prespecified administrative censoring limit for the primary analysis. In the competing-risks models, catheter removal without prior CLABSI, PICU discharge with the catheter *in situ*, and death before CLABSI were treated as competing events. The 28-day censoring horizon was chosen to capture the principal PICU catheter-risk period while limiting instability from sparse late follow-up.

### Covariates and time-varying predictors

Baseline covariates were defined at the time of catheter insertion. Patient-level variables included age, weight, sex, and the presence of underlying chronic comorbidities. Age and weight were transformed to z-scores using established pediatric reference standards to facilitate comparability across the age spectrum. Illness-severity and support variables at insertion included the use of invasive mechanical ventilation, vasoactive or inotropic infusions, and the presence of documented hypotension or multi-organ dysfunction, as recorded in the electronic medical record. Catheter-level variables included anatomic insertion site (femoral, right internal jugular, left internal jugular, subclavian), laterality (right vs. left), and catheter characteristics (e.g., number of lumens).

For the time-to-event analyses, the dataset was reshaped into a patient–day structure, with one record per catheter-day from insertion until CLABSI, catheter removal, PICU discharge, death, or 28 days of dwell. Catheter dwell time was categorized *a priori* into three phases (0–14, 15–22, and ≥23 days), with 0–14 days serving as the reference, based on clinical plausibility and exploratory inspection of the daily hazard. We acknowledge that categorizing dwell time may reduce information and statistical efficiency relative to modeling time as a continuous variable and may accentuate apparent threshold effects. However, given the small number of CLABSI events, a phase-based representation was selected to balance clinical interpretability with model stability. The selected phase boundaries should therefore be interpreted as data-informed clinical risk strata within this cohort rather than as exact biological cutoffs.

Time-varying clinical predictors were derived from routine laboratory and vital-sign measurements. For each catheter-day, we extracted the presence of fever and the most recent white blood cell (WBC) count and C-reactive protein (CRP) measured in the preceding 24 h. Fever was treated as a binary indicator (present vs. absent). WBC and CRP were standardized and entered as continuous variables scaled by their cohort standard deviation. To preserve temporal ordering, these variables were lagged by one day (i.e., the value measured during the previous 24 h predicted the risk of CLABSI on the subsequent day). Indicator variables were used to flag days on which no new measurement was obtained, allowing the model to distinguish between true normal values and missingness.

Total parenteral nutrition (TPN) exposure was handled as a time-varying binary covariate, updated daily to reflect whether the patient was receiving TPN on that catheter-day. Thus, the final models incorporated both fixed baseline characteristics (e.g., age, weight z-score, comorbidities, insertion site) and dynamically updated predictors (fever, WBC, CRP, and TPN) to capture evolving CLABSI risk over the life span of each catheter.

### Statistical analysis

We summarized patient and catheter characteristics using medians with interquartile ranges (IQRs) or counts with percentages, as appropriate. CLABSI incidence was expressed as the number of events per 1,000 catheter-days with 95% confidence intervals (CIs). For the primary analysis, each catheter was converted into patient-day records from insertion until the earliest of CLABSI, catheter removal, PICU discharge, death, or 28 days of dwell, and all regression-based estimates reported in the manuscript were derived within this prespecified analytic window. The binary outcome for each day was the occurrence of a first CLABSI on that day. We then fitted a discrete-time pooled-logistic regression model to estimate adjusted odds ratios (aORs) for daily CLABSI risk. To reflect the anticipated non-linear relationship with dwell time, we categorized catheter-days into three phases (<14, 15–22, and ≥23 days) based on clinical plausibility and exploratory plots. Time-varying covariates (fever, leukocytosis, C-reactive protein) were lagged by 24 h (TTE–1) to preserve temporal ordering, and corresponding indicators captured days when measurements were missing.

Given the small number of CLABSI events and the relatively large number of candidate predictors, we applied ridge regularization to the pooled-logistic model. The penalty parameter *λ* was selected by minimizing the Bayesian Information Criterion. This approach shrinks coefficients toward zero and stabilizes estimates in the presence of multicollinearity and sparse data. We report exponentiated coefficients as aORs with 95% CIs, interpreting them as approximations to daily hazard ratios in the discrete-time framework. To account for competing events, we fitted Fine–Gray subdistribution hazard models using a parsimonious covariate set that included the principal dwell-time phase variables, key time-updated clinical predictors, and insertion-site contrasts, while treating catheter removal, PICU discharge, or death as competing risks. This more compact specification was chosen to improve model stability in the setting of a small number of outcome events. Sub distribution hazard ratios (sHRs) with 95% CIs were used to compare cumulative incidence functions across dwell-time phases and insertion sites. As sensitivity analyses, we fitted Firth-penalized and ridge-penalized Cox proportional hazards models for time to CLABSI, with catheter removal, discharge, or death censored, and we assessed proportional hazards assumptions using Schoenfeld residuals and visual inspection of log–log survival plots. Results of these diagnostics were summarized descriptively and are not shown as separate figures.

Model discrimination was assessed using Harrell's C-index with optimism correction via bootstrap resampling [(e.g., 1,000) samples]. Calibration was examined by plotting observed vs. predicted CLABSI probabilities across deciles of predicted risk and by calculating the daily-level Brier score. To explore the robustness of anatomic site effects, we repeated the primary pooled-logistic analysis in leave-one-site-out sensitivity analyses, sequentially excluding each insertion site. All analyses were performed using R (version 4.3.3; packages survival, riskRegression, coxphf, glmnet) and Python 3.11 (lifelines, scikit-survival, statsmodels). Two-sided *p* < .05 was considered statistically significant. Figures were generated with matplotlib and ggplot2 for publication-ready visualization.

## Results

### Study cohort and CLABSI incidence

Between January 2020 and December 2022, 300 central venous catheter (CVC) insertions in children admitted to the PICU were screened. After applying eligibility criteria, 270 central venous catheters contributed to the final analytic cohort (Figure 1), comprising 4,304 catheter-days, with 13 confirmed central-line–associated bloodstream infections. These 270 catheter episodes represented PICU admissions during the study period, with repeat observations counted only when a child was readmitted on a separate occasion and required a new catheter. Because the analytic unit was the catheter episode, the number of unique children was not assumed to be identical to the number of catheter episodes. Among the included cohort, the median age was 12 months [interquartile range (IQR) 3–48] and the median weight was 7 kg (IQR 4–14); 50.4% were female. The right internal jugular vein was the most common insertion site (57.4%), followed by the left internal jugular vein (21.1%), (Supplementary Figure S1) and the median catheter dwell time was 7 days (IQR 5–9). Comorbidities were present in 61.4% of patients, and 33.0% had fever at line insertion. At baseline, the median white blood cell count was 10.68 × 10³/µL (IQR 7.36–15.45), platelet count 258 × 10³/µL (IQR 156–372), neutrophils 5.96 × 10³/µL (IQR 3.55–9.17), and C-reactive protein 22.5 mg/L (IQR 4.6–56.8). Inotropic support was required in 18.0% of patients, 60.4% were mechanically ventilated, 37.9% received total parenteral nutrition (TPN), and 6.4% developed multi-organ dysfunction syndrome (MODS). The median PICU length of stay was 16 days (IQR 4–21) and the median hospital length of stay was 27 days (IQR 12–64.8); overall mortality was 28.6%. These baseline patient and catheter characteristics are summarized in Supplementary Table S1. Across the 270 catheter observations in the cohort, microbiological isolates were uncommon. The most frequently isolated organisms were coagulase-negative staphylococci (*n* = 5, 1.85%) and Klebsiella pneumoniae (*n* = 4, 1.48%). Acinetobacter baumannii, Pseudomonas aeruginosa, Stenotrophomonas maltophilia, and Candida species were each isolated once (*n* = 1, 0.37% each). The distribution of causative organisms is shown in Supplementary Figure S1.

Over 4,304 central-line days, 13 CLABSIs occurred, corresponding to a crude infection rate of 3.0 per 1,000 central-line days. Using the Saudi national surveillance benchmark of 2.57 CLABSIs per 1,000 central-line days, the expected number of infections in this cohort was approximately 11.1. The resulting standardized infection ratio (SIR) was therefore 1.17 (approximately 1.18), indicating that the observed CLABSI incidence in our PICU was modestly higher than the benchmark based on national reference data.

### Temporal phases and anatomic determinants of CLABSI

**Figure 2:**
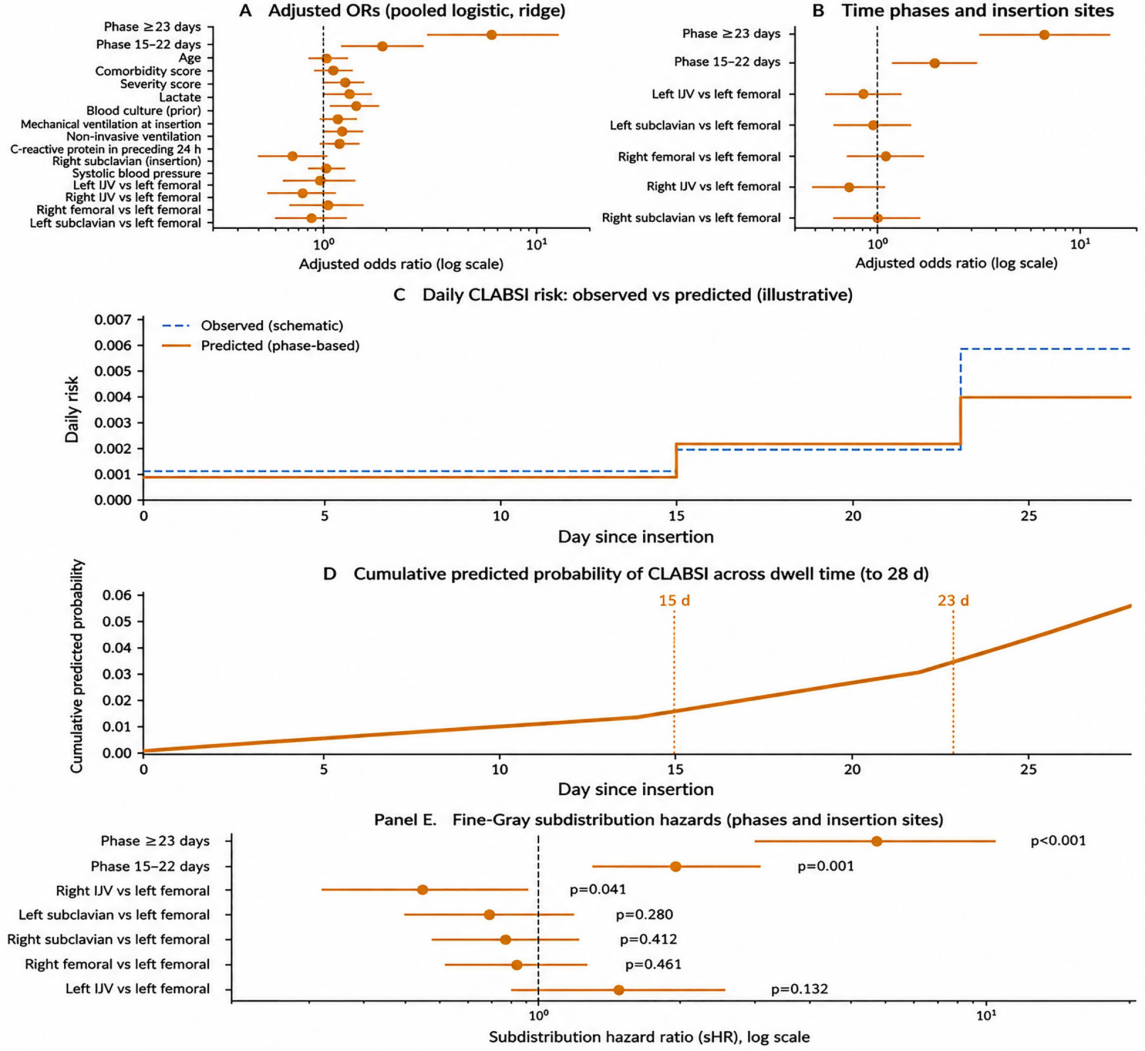
Temporal dynamics of CLABSI risk in PICU patients during the 28-day analytic follow-up period. **(A)** Adjusted odds ratios from the pooled-logistic ridge model for daily CLABSI risk. **(B)** Focused adjusted odds ratios for dwell-time phases and insertion-site contrasts. **(C)** Observed and illustrative phase-based predicted daily CLABSI risk across time since insertion. **(D)** Cumulative predicted probability of CLABSI across dwell time, with vertical dotted lines marking the phase boundaries at 15 and 23 days. **(E)** Fine–Gray subdistribution hazard ratios for CLABSI according to dwell-time phase and insertion site. All time-based panels are presented within the prespecified 28-day administrative follow-up window used for the primary analysis. Site effects should be interpreted as associations within this cohort, not as definitive causal effects.

Survival-time modelling delineated a distinct three-phase hazard pattern for CLABSI (Figures 2A–D). In the ridge-penalized pooled-logistic model, using catheter-days 0–14 as the reference, the adjusted odds of CLABSI increased sharply during days 15–22 (aOR 4.33; 95% CI 1.89–9.92; *p* = 0.001) and were highest for dwell time ≥23 days (aOR 11.71; 95% CI 3.22–42.62; *p* < 0.001) (Table 1; Figures 2B,C). These estimates approximate the daily hazard ratios in the discrete-time framework and confirm a late-phase hazard inflection beginning around the third week of catheter dwell. Anatomic site was also associated with infection risk. After adjustment for dwell phase, time-updated covariates, and baseline confounders, right internal jugular (RIJ) insertion was associated with lower adjusted odds of CLABSI relative to femoral access (aOR 0.34; 95% CI 0.14–0.81; *p* = 0.015) (Table 1; Figure 2B). Other jugular and subclavian sites did not show statistically significant differences compared with femoral in the primary model, although point estimates were directionally consistent with lower risk.

**Table 1:** Adjusted odds ratios (≈daily hazard ratios) for CLABSI from the pooled-logistic ridge model.

Variable	OR	95% CI	Coef	SE	*P* value
Phase 15–22 days	4.33	1.89–9.92	1.47	0.42	0.001
Phase ≥ 23 days	11.71	3.22–42.62	2.46	0.66	<0.001
Fever in preceding 24 h	3.00	0.94–9.52	1.10	0.59	0.07
TPN (exposure)	1.15	0.36–3.67	0.14	0.59	0.56
White blood cell counts in preceding 24 h (per SD)	1.21	0.63–2.32	0.19	0.38	0.61
C-reactive protein in preceding 24 h (per SD)	1.18	0.59–2.36	0.17	0.40	0.66
Age (z-score)	0.91	0.39–2.14	−0.09	0.44	0.83
Weight (z-score)	0.91	0.38–2.22	–0.09	0.45	0.84
Inotrope use at insertion	0.82	0.25–2.68	−0.20	0.60	0.75
Mechanical ventilation at insertion	0.77	0.33–1.81	−0.26	0.43	0.56
Right internal jugular vs. femoral	0.34	0.14–0.81	−1.07	0.44	0.015

Table 1 shows adjusted odds ratios from the pooled-logistic ridge regression model with daily time intervals. Time-updated predictors were defined using values recorded during the preceding 24 h. White blood cell count and C-reactive protein were standardized and entered per cohort standard deviation. Right internal jugular access was associated with lower adjusted odds compared with the femoral reference site.

**Figure 3:**
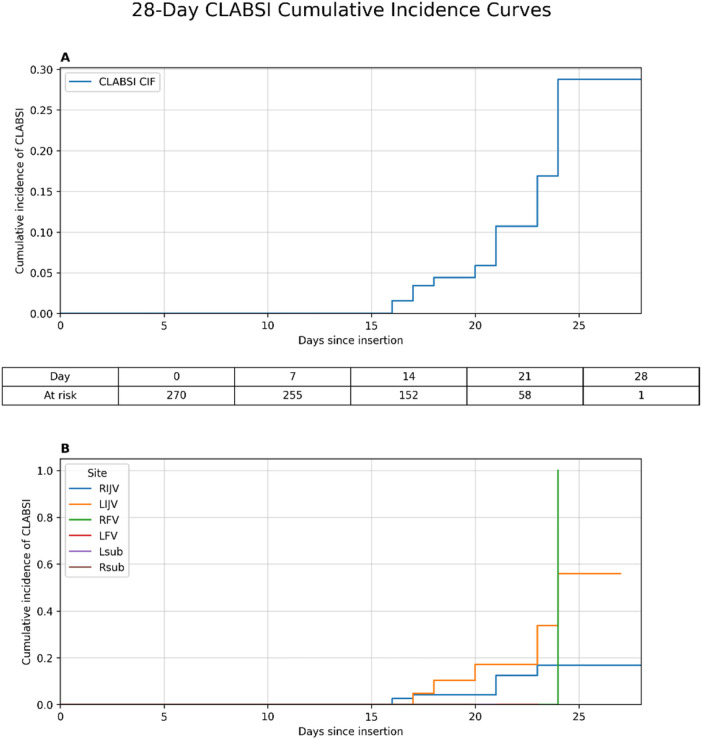
Twenty-eight-day cumulative incidence curves for CLABSI. **(A)** Overall cumulative incidence of first CLABSI during the 28-day analytic follow-up period after catheter insertion. The table below indicates the number of catheter episodes at risk at days 0, 7, 14, 21, and 28. **(B)** Cumulative incidence of first CLABSI stratified by insertion site over the same 28-day follow-up period. RIJV, right internal jugular vein; LIJV, left internal jugular vein; RFV, right femoral vein; LFV, left femoral vein; Lsub, left subclavian vein; Rsub, right subclavian vein. All curves shown in this figure are presented within the 28-day administrative follow-up window used for the primary analysis.

In Fine–Gray subdistribution hazard models, which treated catheter removal, PICU discharge, and death as competing risks, the cumulative incidence of CLABSI displayed a similar phase-dependent pattern. Because of the small number of CLABSI events, the competing-risks analysis used a more parsimonious covariate specification than the primary pooled-logistic model. Relative to dwell time <14 days, subdistribution hazard ratios (sHRs) were 3.9 (95% CI 1.6–9.6) for days 15–22 and 7.8 [95% CI (insert CI if available); *p* < 0.001] for dwell time ≥23 days (Table 2; Figure 2E and Figures 3A,B). Right internal jugular access remained associated with lower cumulative incidence compared with femoral access (sHR ≈ 0.4; *p* ≈ 0.04), consistent with the lower-risk association observed in the pooled-logistic model (Table 2; Figure 2E).

**Table 2:** Fine–gray sub-distribution hazard ratios for CLABSI with competing events (line removal, discharge, death).

Variable	sHR	95% CI	*P* value
Phase 15–22 days	3.9	1.6–9.6	0.002
Phase ≥23 days	7.8	2.5–24.7	<0.001
Fever in preceding 24 h	2.9	1.12–7.3	0.03
Total parenteral nutrition exposure	1.8	0.9–3.5	0.08
White blood cell count in preceding 24 h (per SD)	1.2	0.6–2.4	0.58
C-reactive protein in preceding 24 h (per SD)	1.1	0.5–2.3	0.71
Right internal jugular vs. femoral	0.42	0.17–0.96	0.04

Table 2 presents Fine–Gray subdistribution hazard ratios for CLABSI in the presence of competing events (catheter removal, PICU discharge, or death). To preserve model parsimony in a sparse-event setting, the competing-risks model included dwell-time phases, selected time-updated clinical predictors, and insertion-site contrasts rather than the full pooled-logistic covariate set. Time-updated predictors were defined using values recorded during the preceding 24 h. White blood cell count and C-reactive protein were standardized and entered per cohort standard deviation.

### Time-updated clinical predictors

Time-varying clinical variables provided additional discrimination of daily CLABSI risk. In the pooled-logistic ridge model, fever during the preceding 24 h was associated with a threefold higher adjusted odds of CLABSI (aOR 3.00; 95% CI 0.94–9.52; *p* = 0.07), although the confidence interval included 1.0 (Table 1). Total parenteral nutrition (TPN) exposure on a given day was associated with a modest, non-significant increase in risk (aOR 1.15; 95% CI 0.36–3.67; *p* = .56). Standardized white blood cell count and C-reactive protein levels lagged by 24 h showed aORs of 1.21 (95% CI 0.63–2.32; *p* = 0.61) and 1.18 (95% CI 0.59–2.36; *p* = 0.66), respectively, indicating a numerically higher but statistically imprecise risk (Table 1). Age, weight (both as z-scores), mechanical ventilation, and inotrope use at insertion were not significantly associated with daily CLABSI odds in the penalized model. Taken together, these findings suggest that the highest-risk scenario for CLABSI occurs when prolonged dwell time (≥15 days, particularly ≥23 days) coincides with femoral access and recent fever, with TPN and inflammatory markers providing additional, albeit imprecise, risk information (Figures 2A–D and Figures 3A,B).

### Model performance, calibration, and sensitivity analyses

**Figure 4:**
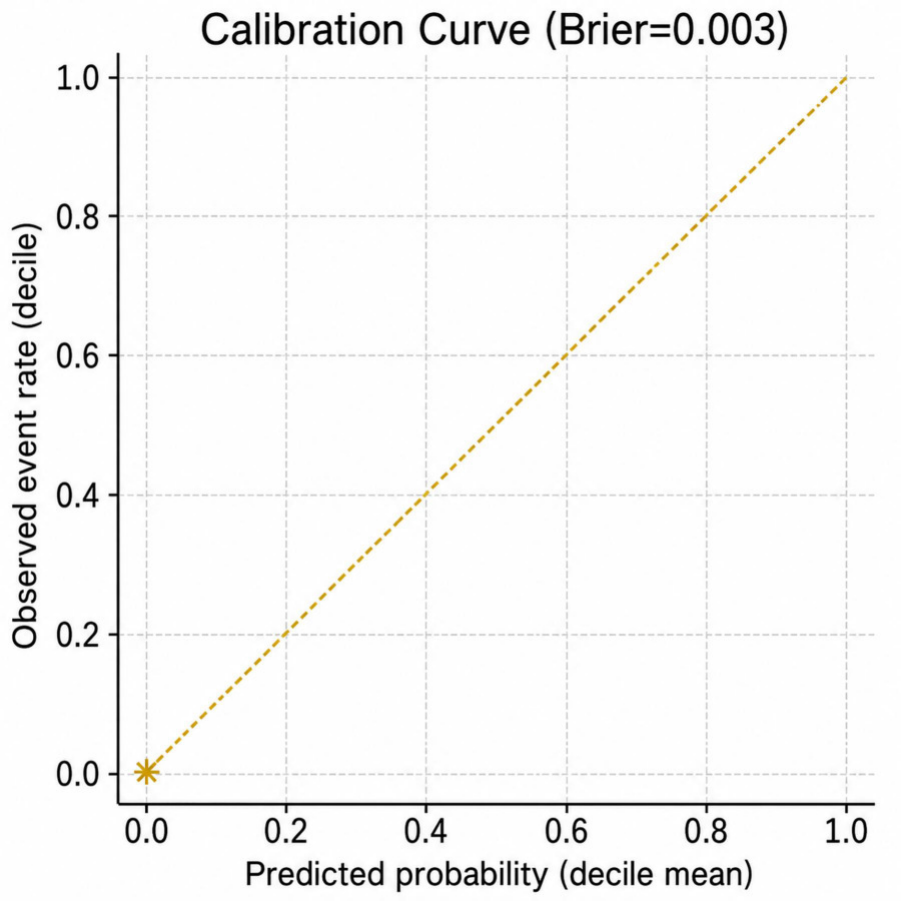
Calibration curve demonstrating agreement between predicted and observed probabilities for daily CLABSI risk. The figure depicts model calibration across deciles of predicted risk derived from the pooled-logistic ridge regression model. The *x*-axis represents the mean predicted probability of central line–associated bloodstream infection (CLABSI) within each decile, and the *y*-axis shows the corresponding observed event rate. The dashed 45° line denotes perfect calibration. Each plotted point reflects the observed infection frequency relative to predicted risk, showing close alignment across the risk spectrum. The model demonstrated excellent calibration with a Brier score of 0.003, indicating minimal deviation between predicted and actual probabilities.

The primary pooled-logistic ridge model showed promising apparent internal performance, although these estimates should be interpreted cautiously given the small number of outcome events. In 1,000 bootstrap resamples, the apparent and optimism-corrected Harrell's C-index were both 0.78 (95% CI 0.70–0.86), indicating good separation between catheter-days with and without subsequent CLABSI (Table 3). The bias-corrected calibration slope was 0.93 (95% CI 0.82–1.04) and the intercept 0.00 (95% CI −0.07–0.05), consistent with near-ideal calibration (Table 3). The overall Brier score was 0.003, reflecting very good predictive accuracy at the daily level given the low absolute event rate (Table 3).The decile-wise calibration and observed event rates are shown in Figure 4 and Supplementary Table S2.

**Table 3:** Model validation metrics: discrimination and calibration (bootstrap-corrected).

Metric	Apparent	Bias-Corrected	95% CI
C-index	0.78	0.78	0.70–0.86
Calibration slope	0.96	0.93	0.82–1.04
Calibration intercept	–0.02	0.00	–0.07–0.05
Brier score	0.003	0.003	–

Internal validation results from 1,000 bootstrap iterations assessing discrimination and calibration of the pooled-logistic ridge model for CLABSI prediction. The bias-corrected C-index and calibration slope indicate stable performance with minimal optimism. CI, confidence interval.

Sensitivity analyses supported the robustness of the main findings. Ridge- and Firth-penalized Cox proportional hazards models yielded effect estimates for dwell-time phases and right internal jugular insertion that were directionally concordant with the discrete-time and Fine–Gray models. Visual inspection of Schoenfeld residuals and log–log survival plots did not reveal major violations of the proportional hazard's assumption for these key covariates (diagnostic plots not shown). A leave-one-site-out ridge analysis, in which each anatomic site category was sequentially excluded and the model re-estimated, showed stable associations for dwell-time phases and a consistently lower-risk pattern for jugular relative to femoral access, with only modest widening of confidence intervals (Supplementary Figure S2).

## Discussion

This 3-year time-to-event analysis shows a clear temporal pattern of central-line–associated bloodstream infection (CLABSI) risk in critically ill children. Risk is low in the first two weeks, increases between days 15 and 22, and rises sharply after day 23. This pattern, confirmed by ridge-penalized and Fine–Gray competing-risk models, indicates that CLABSI risk changes over time rather than remaining constant across the lifespan of a catheter. Our findings offer a practical guide for time-based clinical decisions instead of relying on arbitrary dwell-time thresholds.

### Temporal risk and implications for line stewardship

The stepwise increase in daily odds—approximately fourfold during days 15–22 and elevenfold beyond day 23—identifies a realistic window for intervention. These results support time-triggered “line-necessity reviews” at days 14 and 21 that can be built into standard bundle workflows. Rather than promoting routine catheter exchanges, which are explicitly discouraged by current guidelines (4, 33), these checkpoints serve as structured opportunities to remove nonessential lines before patients enter the highest-risk phase. As shown in Figure 2D, the cumulative predicted probability of CLABSI increases steeply after day 23, making these thresholds important points for reassessment and potential removal. Emerging multicenter pediatric data support a similar pattern, with dwell-time–related hazard increases around the third week of catheterization even after adjustment for illness severity and insertion site. Taken together, these findings support the idea that dwell time should be treated as a modifiable, time-sensitive risk factor within central-line stewardship programs. Importantly, the proposed day-14 and day-21 review points should not be interpreted as replacing routine daily assessment of line necessity. In our PICU, catheter necessity is reviewed during daily goal-directed rounds, in which the care team explicitly asks whether the line remains clinically necessary. Rather, the day-14 and day-21 time points represent additional time-triggered stewardship checkpoints derived from the observed phase-specific rise in CLABSI risk in this cohort. Earlier reassessment may be warranted in individual patients based on insertion circumstances, dressing compromise, suspected contamination, catheter dysfunction, or emerging clinical concern for infection.

### Comparison with prior pediatric evidence

Our results are consistent with previous pediatric studies showing that longer catheter dwell time independently predicts CLABSI across central-line types. In neonatal and infant populations, extended catheter duration has repeatedly been identified as a major driver of infection risk. In a large prospective infant cohort, Greenberg et al. reported that central venous catheter dwell time was strongly associated with bloodstream infection, especially for tunneled lines (9). Advani et al. found that in neonates with peripherally inserted central catheters (PICCs), infection risk rose steadily during the first two weeks and remained elevated thereafter (25). More recent multicenter work supports these temporal patterns. A national Chinese cohort of umbilical venous catheters (UVCs) reported a dwell-time–dependent increase in CLABSI incidence beyond 20 days (34), while studies by Pitiriga et al. and Buttera et al. described similar inflection points for pediatric CVCs and PICCs, with risk tripling after 21 days of use (35, 36) A 2024 meta-analysis by Li et al. pooled 27 pediatric CLABSI studies and confirmed prolonged catheterization as an independent risk factor (summary OR = 1.19, 95% CI 1.07–1.33) (37), in keeping with our phase-specific hazard increase. Together, these data and our findings support a biologically plausible pathway in which extended intravascular exposure promotes biofilm maturation, endothelial injury, and microbial colonization, leading to the accelerating infection risk seen after the third week of catheterization.

### Insertion site considerations

In our cohort, femoral access accounted for a disproportionate share of CLABSI episodes, while no infections were observed among subclavian catheters. These observations must be interpreted cautiously given the small number of events and potential confounding by indication. However, right internal jugular access consistently showed lower adjusted odds and lower subdistribution hazards relative to femoral placement in this cohort. These findings should be interpreted cautiously given the small number of events and the possibility of residual confounding related to insertion context, catheter handling, and clinical indication. This pattern is similar to adult data supporting subclavian access for infection prevention (31), but differs from several pediatric reports that describe similar risks across sites when maintenance bundles are strictly followed (9, 11, 38).

In a pediatric intensive care study using inverse-probability treatment weighting, femoral and jugular non-tunneled CVCs had comparable infection rates after statistical adjustment, suggesting that high-quality line care can offset some anatomical differences (39). In contrast, larger pediatric series by Gowardman et al. and Moriarty et al. documented higher colonization and bloodstream infection rates with femoral catheters compared with jugular or subclavian sites (25, 40). A multicenter study published in *Antimicrobial Resistance and Infection Control* in 2020 also found that femoral catheterization was independently associated with colonization by multidrug-resistant organisms compared with internal jugular or subclavian access. Similarly, a 2024 Bayesian network meta-analysis by Guerra et al. identified jugular access as the most protective site in children, although equipoise remains in infants pending results of the PRECiSE randomized trial (41). Emerging registry analyses from 2025 are consistent with these observations, suggesting that jugular insertion does not increase infection risk and may add safety when performed under ultrasound guidance and within bundle-compliant settings. Until larger pediatric trials provide definitive evidence, insertion-site choice should be individualized, clearly justified in the medical record, and tracked through unit-level dashboards that link dwell time, maintenance compliance, and infection outcomes.

All catheter sites in our PICU were managed under the same high-compliance institutional prevention bundle, with documented overall adherence of 98%–99% during the study period. This makes broad unit-level nonadherence an unlikely sole explanation for the observed site-related differences. However, because our retrospective dataset did not permit site-specific assessment of catheter-level adherence, access frequency, dressing disruption, or other maintenance-related practices, residual confounding remains possible. In addition, urgency of placement (emergent vs. elective) was not consistently captured for either femoral or non-femoral lines and could not be modeled directly. Accordingly, the observed insertion-site associations should not be interpreted as purely anatomical effects independent of insertion context and bedside practice.

### Role of parenteral nutrition and inflammatory markers

Total parenteral nutrition (TPN) remains a strong, modifiable risk factor for CLABSI, likely due to lipid-rich infusions and frequent manipulation of the catheter hub (11, 42–45), targeted TPN sub-bundle—using a dedicated lumen, minimizing hub access, and involving pharmacists in indication review—should accompany PN initiation. Our data show that the combination of fever, leukocytosis, and TPN use substantially increases the short-term probability of infection, highlighting their value as bedside warning signals. When these features occur together, a structured bedside checklist that includes cultures, a bundle audit, and a line-necessity review is consistent with sepsis guidance and can be reinforced by electronic alerts (11).

### Microbiological and mechanistic correlates

Gram-negative organisms were the most frequent pathogens in this cohort, and Candida species also contributed meaningfully. This profile is similar to global PICU data (46–48). Empiric therapy in this setting should therefore include early Gram-negative coverage, with prompt narrowing once susceptibilities are available. From a mechanistic perspective, parenteral lipid emulsions may promote Candida germination and enhance biofilm formation within catheter hubs (49–51), which explains the strong association between lipid exposure and fungal catheter infections. These data support restricting antifungal prophylaxis to clearly defined ultra–high-risk groups. Consistent with recent pediatric stewardship recommendations (52–54), we favour an approach that maintains vigilance for invasive candidiasis but uses empiric antifungals judiciously to limit resistance and toxicity.

### Clinical and health-system consequences

Each CLABSI episode carries major clinical and financial consequences. Prior work suggests that a single infection can prolong hospitalization by about 20 days and add roughly US$50 000 in attributable costs (3, 4). Beyond costs, CLABSI disrupts families, delays recovery, and erodes trust in care. These realities justify investment in electronic health record (EHR)–embedded alerts and real-time dashboards that support proactive prevention instead of retrospective audit alone. Time-to-event modeling, as applied here, can inform these tools by translating daily infection probabilities into clear thresholds for action.

### Methodological strengths and sensitivity analyses

Our analysis combined ridge-penalized pooled-logistic models with Fine–Gray and cause-specific Cox approaches to estimate daily risk while reducing small-event bias. The primary pooled-logistic ridge model showed promising apparent internal performance, although these estimates should be interpreted cautiously given the small number of outcome events. Internal bootstrap validation (C-index = 0.78) showed good discrimination, and bias-correction analysis supported the stability of the coefficients. Sensitivity analyses that varied the administrative censoring time and left out individual insertion sites produced similar dwell-phase and site effects, reinforcing the robustness of the findings. Although our event count was modest, these checks support the reliability of the main inferences. The analytic framework followed TRIPOD and STROBE extensions for prediction modeling and may serve as a practical template for multicenter CLABSI surveillance studies (55).

### Limitations and future directions

The main limitation of this study is the small number of CLABSI events (*n* = 13) relative to the number of modeled predictors and parameters. Although ridge penalization and bootstrap-based internal validation were used to reduce overfitting and stabilize estimates, these methods do not fully overcome the methodological limitations of sparse-event prediction modeling. With few outcome events, regression coefficients may remain unstable, apparent model performance may be optimistic, and calibration estimates may remain imprecise despite shrinkage. The present model should therefore be interpreted as an internally derived, exploratory risk model intended to characterize temporal and anatomic patterns in this cohort, rather than as a finalized clinical prediction tool ready for direct transport to other settings (56). Our findings also apply primarily to non-tunneled CVCs and may not generalize to tunneled or implanted devices. Residual confounding by indication remains possible despite adjustment. Although documented overall central-line bundle compliance was consistently high (98%–99%) during the study period, compliance was assessed through routine electronic documentation review rather than prospective catheter-level observation of each bundle element. We therefore could not directly compare adherence between insertion and maintenance practices, nor could we assess whether compliance differed by insertion site, particularly for femoral vs. non-femoral lines. Similarly, emergent vs. elective line placement was not uniformly documented and could not be evaluated as a covariate. These factors may have contributed to residual confounding in the observed temporal and site-related associations.

A further limitation is the categorization of catheter dwell time into three phases. While this phase-based approach improved clinical interpretability and facilitated translation of the findings into pragmatic line-stewardship checkpoints, categorizing a continuous or time-varying predictor is known to reduce information, diminish statistical efficiency, and potentially exaggerate threshold effects that are not truly discrete (57, 58). In addition, when cut points are influenced by the observed data, they may introduce optimism, reduce reproducibility, and perform less well in new datasets (57, 59). Accordingly, the dwell-time categories used here should be interpreted as data-informed clinical risk strata within this cohort rather than biologically exact or universally transportable transition points. We adopted this simplified representation to balance interpretability with model stability in a sparse-event setting; however, future larger multicenter studies with greater event counts should model dwell time more flexibly, for example using restricted cubic splines or fractional polynomials, to better define the continuous shape of CLABSI hazard over catheter life (60).

## Conclusion

In summary, CLABSI risk in the pediatric intensive care unit is time-dependent and phase-specific rather than static. The transition into the third week of catheterization marks a key inflection point for infection risk. Fever, leukocytosis, and parenteral nutrition emerge as important time-updated markers that, when combined with electronic monitoring and standard bundles, can support earlier preventive action. Integrating data-driven, time-triggered line reviews into routine care appears to be a feasible and evidence-based strategy to reduce CLABSI incidence, shorten hospital stays, and improve outcomes for critically ill children.

## Data Availability

The raw data supporting the conclusions of this article will be made available by the authors, without undue reservation.
