# Supplementary material for: Temporal and anatomic determinants of central-line–associated bloodstream infection risk in a tertiary PICU: a 3-year time-to-event and competing-risk analysis
Source: Front Pediatr. 2026 Jun 12;14:1763433. doi: 10.3389/fped.2026.1763433 (PMC13306186; doi:10.3389/fped.2026.1763433)
Supplement: Supplementary file 1 [file Datasheet1.docx]

Temporal and Anatomic Determinants of Central-Line–Associated Bloodstream Infection (CLABSI) Risk in a Tertiary PICU: A 3-Year Time-to-Event and Competing-Risk Analysis.

*Supplementary Documents:*

Table S1: Base Line Characteristics of our Cohorts.

| Level | Overall |
| --- | --- |
| n | 270 |
| **Age (median [IQR])** | 12.00 [3.00, 48.00] |
| **Gender n (%)** |  |
| *Female* | 136 (50.4) |
| *Male* | 134 (49.6) |
| **Wt (median [IQR])** | 7.00 [4.00, 14.00] |
| **Insertion Site (%)** |  |
| *Left Femoral Vein (LFV)* | 11 (4.1) |
| *Left Internal Jugular Vein (LIJV )* | 57 (21.1) |
| *Left Subclavian ( Lsub )* | 7 ( 2.6) |
| *Right Femoral Vein (RFV )* | 33 (12.2) |
| *Right Internal Jugular Vein (RIJV)* | 155 (57.4) |
| *Right Subclavian (Rsub )* | 7 ( 2.6) |
| **CLABSI (%)** |  |
| *Positive* | 13 (4.8) |
| *Negative* | 257 (95.2) |
| **Co-Morbidity n (%)** |  |
| *Yes* | 164 (61.4) |
| *No* | 103 (38.6) |
| **Fever n (%)** |  |
| *Yes* | 88 (33.0) |
| *No* | 179 (67.0) |
| **WBC (median [IQR])** | 10.68 [7.36, 15.45] |
| **Platelet (median [IQR])** | 258.00 [156.00, 372.00] |
| **Neutrophil (median [IQR])** | 5.96 [3.55, 9.17] |
| **CRP (median [IQR])** | 22.50 [4.59, 56.83] |
| Inotropes n (%) |  |
| *Yes* | 48 (18.0) |
| *No* | 219 (82.0) |
| **Multiorgan Dysfunction Syndrome (MODS)(%)** |  |
| *Yes* | 17 ( 6.4) |
| *No* | 249 (93.6) |
| **Mechanical Ventilation (MV %)** |  |
| *Yes* | 162 (60.4) |
| *No* | 106 (39.6) |
| **TPN n (%)** |  |
| *Yes* | 102 (37.9) |
| *No* | 167 (62.1) |
| **Length of PICU stay (LOPICUS) (median [IQR])** | 16.00 [4.00, 21.00] |
| **Length of Hospital Stay (LOHS) (median [IQR])** | 27.00 [12.00, 64.75] |
| **Outcome (%)** |  |
| *Died* | 77 (28.6) |
| *Survive* | 192 (71.4) |


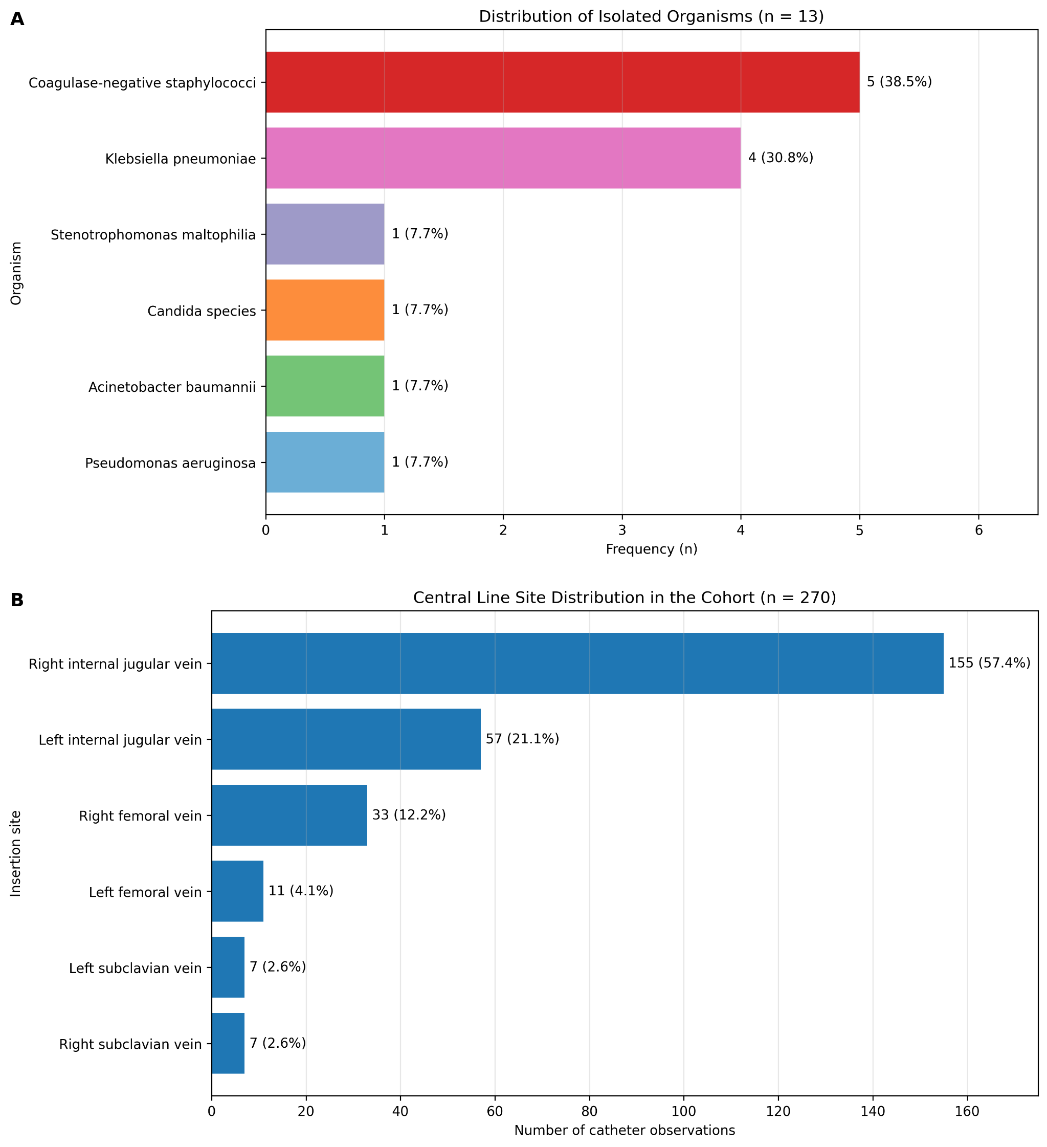


Figure S1. Distribution of causative organisms and catheter insertion sites in the study cohort.(A) Frequency distribution of organisms isolated from the 13 CLABSI episodes. Coagulase-negative staphylococci were the most common pathogens (5/13, 38.5%), followed by *Klebsiella pneumoniae* (4/13, 30.8%). *Acinetobacter baumannii,* *Pseudomonas aeruginosa, Stenotrophomonas maltophilia,* and *Candida spp*. were each identified in 1 episode (7.7%). No polymicrobial CLABSI episodes were observed.(B) Distribution of central venous catheter insertion sites among the 270 catheter observations included in the analytic cohort. The right internal jugular vein was the most common site (155/270, 57.4%), followed by the left internal jugular vein (57/270, 21.1%) and right femoral vein (33/270, 12.2%). Left femoral, left subclavian, and right subclavian sites were less frequently used.

Table S2: Calibration Results by Decile of Predicted CLABSI Risk

| **Decile** | | **n** | **Mean Predicted Probability** | **Observed Event Rate (%)** |
| --- | --- | --- | --- | --- |
| 1 | 412 | | 0.00087 | 0.00 |
| 2 | 407 | | 0.0017 | 0.00 |
| 3 | 395 | | 0.0020 | 0.00 |
| 4 | 412 | | 0.0023 | 0.00 |
| 5 | 392 | | 0.0025 | 0.00 |
| 6 | 405 | | 0.0028 | 0.00 |
| 7 | 403 | | 0.0034 | 0.00 |
| 8 | 410 | | 0.0048 | 0.00 |
| 9 | 394 | | 0.0076 | 1.27 |
| 10 | 404 | | 0.016 | 1.98 |

***Footnote:*** Decile-wise calibration results from the pooled-logistic ridge model for CLABSI prediction. The mean predicted probability corresponds to the average estimated daily infection risk within each decile of predicted values; the observed event rate represents the proportion of actual CLABSI events within the same.


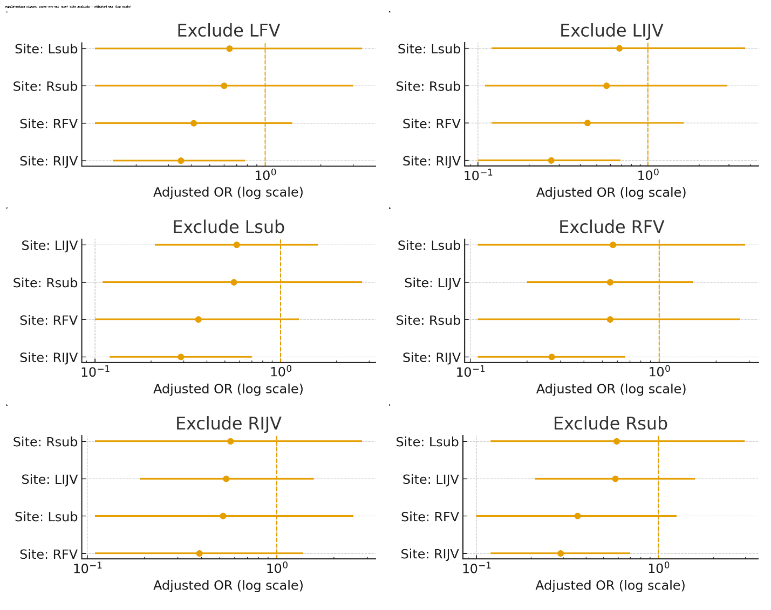


Figure S2: Leave-one-out site sensitivity analysis showing adjusted odds ratios (log scale) for central line insertion sites from ridge-penalized pooled-logistic regression models. Each panel represents model re-estimation after exclusion of one site category (e.g., femoral, jugular, or subclavian). Horizontal lines denote 95% confidence intervals. The stability of directionality across all panels confirms robustness of insertion-site effects to data exclusion.
